# Evaluation of Medetomidine Dose Variations on Tiletamine-Zolazepam and Tramadol Anesthetic Combination in Dogs

**DOI:** 10.3390/ani15233419

**Published:** 2025-11-26

**Authors:** Donghwi Shin, Won-gyun Son, Jong-pil Seo, Min Jang, Inhyung Lee

**Affiliations:** 1 Department of Veterinary Clinical Sciences, College of Veterinary Medicine, Seoul National University, Seoul 08826, Republic of Korea; hwi4010@snu.ac.kr (D.S.); carpeego@snu.ac.kr (W.-g.S.); 2 Department of Veterinary Clinical Sciences, College of Veterinary Medicine, Jeju National University, Jeju 63243, Republic of Korea; jpseo@jejunu.ac.kr; 3 Department of Veterinary Surgery, College of Veterinary Medicine, Kyungpook National University, Daegu 41566, Republic of Korea; jangmin@knu.ac.kr

**Keywords:** α_2_-adrenergic agonist, dissociative, dog, injectable anesthesia, medetomidine, opioid

## Abstract

This study evaluated the effects of varying medetomidine doses on a tiletamine-zolazepam and tramadol anesthetic combination in dogs. The objective was to assess how medetomidine influences anesthesia depth, cardiovascular function, and recovery quality. Dogs were assigned to one of three treatment groups: tiletamine-zolazepam and tramadol without medetomidine (TZT), additional 10 µg/kg medetomidine (TZTM10), or additional 20 µg/kg medetomidine (TZTM20). Each dog received an intramuscular injection of 0.1 mL/kg of the designated solution. The formulation for TZT contained 25 mg/mL tiletamine and zolazepam and 40 mg/mL tramadol, while TZTM10 and TZTM20 received the same solution with additional medetomidine at 100 and 200 µg/mL, respectively. Onset time, anesthetic time, analgesic time, recovery quality, and physiological parameters, including heart rate, blood pressure, respiratory rate, oxygen saturation, and body temperature, were evaluated. Medetomidine extended analgesic time in a dose-dependent manner and improved recovery quality while maintaining stable respiratory function. While TZT provided adequate anesthesia for minor non-invasive procedures, the inclusion of medetomidine in TZTM resulted in enhanced anesthetic stability for simple surgical procedures such as gonadectomy. These findings suggest that TZTM20 is a viable anesthetic protocol, though careful patient monitoring remains essential due to its cardiovascular effects.

## Simple Summary

Veterinary anesthesia often requires combining multiple drugs to achieve safe and effective anesthesia for short procedures in dogs. This study evaluates an anesthetic combination of tiletamine-zolazepam, tramadol, and medetomidine (TZTM), focusing on how different medetomidine doses affect anesthesia depth, analgesia, and cardiovascular stability. The combination excludes highly regulated opioids, making it more accessible and easier to prepare. Three formulations were tested: one without medetomidine and two containing different concentrations. The physiological parameters, including heart rate, blood pressure, respiratory rate, oxygen saturation, analgesic effect, and recovery quality, were assessed. The results indicated that medetomidine dose-dependently prolonged anesthesia and analgesia while transiently reducing heart rate and oxygen saturation. Higher medetomidine doses improved recovery quality, suggesting its suitability for stable and balanced sedation. However, due to its cardiovascular effects, careful patient selection is necessary, particularly in individuals with pre-existing heart conditions. These findings highlight TZTM as a practical alternative for short veterinary procedures, provided that appropriate monitoring is implemented.

## 1. Introduction

Intramuscular (IM) anesthetic protocols offer an alternative to inhalation anesthesia in dogs, particularly under specific clinical conditions. As a result, various studies have focused on optimizing IM anesthesia to improve its safety and efficacy [1,2,3,4,5,6]. However, IM anesthesia presents inherent challenges, including the difficulties of continuous monitoring and maintaining stable plasma drug levels. Consequently, an effective protocol should incorporate a wide safety margin, provide adequate analgesia and muscle relaxation, allow partial reversal when necessary, and ensure rapid induction, sufficient anesthetic duration, and predictable recovery following a single injection [7]. For an IM anesthetic protocol to be considered optimal, it should involve agents that are not only safe but also fast-acting, capable of inducing reliable anesthesia, offering postoperative pain relief, and reversible when needed [8]. Among the drugs commonly used in injectable anesthesia, tiletamine-zolazepam, tramadol, and medetomidine meet these criteria to a certain extent, which has led to multiple studies evaluating their combined use [1,2,5].

Maintaining stable and appropriate anesthesia is essential for surgical procedures performed with IM injection. An anesthetic protocol should ensure a swift onset of unconsciousness while providing muscle relaxation, amnesia, and reflex suppression. Furthermore, adequate analgesia is critical for reducing procedural stress and enhancing patient stability [8,9]. Additionally, the selected anesthetic agents should have a broad safety margin, and the availability of an antagonist is advantageous for controlled recovery. Considering the need for both safety and efficiency in intramuscular anesthesia, selecting drugs specifically suited for this route of administration is essential. Given the requirements for safe and effective IM anesthesia, the combination of tiletamine, zolazepam, tramadol, and medetomidine was selected for its ability to provide multimodal analgesia with several different analgesic mechanisms.

A well-balanced anesthetic protocol often relies on the combination of multiple agents at lower doses rather than a single drug, a strategy known as balanced anesthesia. This approach enhances safety by minimizing the adverse effects associated with high doses of a single anesthetic [10,11]. One such combination, tiletamine–zolazepam–ketamine–xylazine (TKX), was initially developed for feline surgical procedures, such as onychectomy and castration [12], and has since been widely used in high-volume animal shelters and elective surgeries in cats [8,13,14]. However, the TKX combination was primarily formulated for cats and does not fully meet the anesthetic requirements for dogs. The inclusion of ketamine in TKX was intended to modify the standard 1:1 tiletamine–zolazepam ratio to prevent prolonged recoveries caused by higher doses of zolazepam in cats [12]. In dogs, however, ketamine often induces muscle rigidity, leading to rough and extended recovery periods due to the combined effects of tiletamine and ketamine. Although xylazine offers the advantage of reducing drug volume requirements [8,15], advancements in α_2_-adrenergic agonists have led to a decline in its use for small animals, with medetomidine largely replacing it in clinical practice [1]. Another limitation of the TKX combination is the absence of an opioid, which reduces its ability to provide multimodal analgesia. To enhance both sedation and analgesia, medetomidine is often combined with butorphanol, which produces reliable sedative and analgesic effects in dogs [16,17,18]. However, butorphanol’s analgesic efficacy is limited due to its antagonist activity at μ-opioid receptors and agonist activity at κ-opioid receptors [19]. To address this limitation, tramadol was included as a more accessible alternative to butorphanol, particularly in contexts where the use of controlled substances is restricted. Although its analgesic efficacy in dogs is limited, the absence of μ-opioid receptor antagonism and its practical advantages support its adjunctive use as part of a multimodal analgesic strategy.

The objectives of this study were to evaluate and compare the anesthetic and cardiorespiratory effects of varying medetomidine doses in a tiletamine-zolazepam–tramadol combination in dogs, with the aim of identifying the most stable protocol with minimal adverse effects. It was hypothesized that the addition of medetomidine would result in dose-dependent changes in analgesic duration, recovery characteristics, and physiological stability following IM administration of the combination. The findings are expected to contribute to the refinement of IM anesthetic protocols, ultimately improving clinical outcomes in veterinary practice.

## 2. Materials and Methods

### 2.1. Animals

This study was conducted on six healthy male Beagle dogs, all aged three years. The sample size was determined with reference to previous studies in veterinary anesthesia that employed similar objectives [1,2,16,17,18]. Blood analysis confirmed normal liver and kidney function in all subjects. Each dog received all three treatments in a repeated-measures design, with a minimum two-week washout period between trials. The order of treatment administration was varied by drawing lots among animals to minimize potential sequence effects.

All experimental procedures were approved by the Institutional Animal Care and Use Committee of Seoul National University (approval number: SNU-131224-1). All dogs were housed individually in indoor kennels maintained at 22–25 °C. They had free access to water and were fed once or twice daily. Environmental enrichment included supervised leash walks or interactive play with a volunteer group from the College of Veterinary Medicine at least three times per week. Prior to the study, the dogs were fasted for at least 6 h with free access to water. Before anesthesia induction, all dogs underwent a basic physical examination, including an assessment of capillary refill time, mucous membrane color, heart rate (HR), blood pressure (BP), respiratory rate (RR), and body temperature (Temp).

### 2.2. Anesthesia Protocol

Each group received an IM injection of 0.1 mL/kg into biceps femoris with a distinct anesthetic combination:

- TZT: A combination of tiletamine-zolazepam powder (250 mg, Zoletil^®^50, Virbac Co., Ltd., Carros, France), 4 mL of tramadol (50 mg/mL, Toranzin Inj. 50 mg, Samsung Pham Co., Ltd., Hwaseong, Republic of Korea), and 1 mL of sterile water. The final solution contained 25 mg of tiletamine, 25 mg of zolazepam, and 40 mg of tramadol per 1 mL.
- TZTM10: A combination of tiletamine-zolazepam powder (250 mg), 4 mL of tramadol (50 mg/mL), 0.5 mL of medetomidine (1 mg/mL, Domitor^®^, Orion Pharma, Espoo, Finland), and 0.5 mL of sterile water. The final solution contained 25 mg of tiletamine, 25 mg of zolazepam, 40 mg of tramadol, and 100 µg of medetomidine per 1 mL.
- TZTM20: A combination of tiletamine-zolazepam powder (250 mg), 4 mL of tramadol (50 mg/mL), 1 mL of medetomidine (1 mg/mL). The final solution contained 25 mg of tiletamine, 25 mg of zolazepam, 40 mg of tramadol, and 200 µg of medetomidine per 1 mL.

### 2.3. Experimental Procedures

To facilitate arterial catheterization, lidocaine (0.2 mL/kg, Daihan Lidocaine HCl Hydrate Inj. 2%, Daihan Pharm Co., Ltd., Seoul, Republic of Korea) was infiltrated into the metatarsal region of the hind limb for local anesthesia. A 22-gauge intravenous (IV) catheter (3S-Cath, Dukwoo Medical Co., Ltd., Hwaseong, Republic of Korea) was placed in the dorsal pedal artery to enable invasive BP monitoring. Following catheter placement, each dog was allowed to stabilize for 30 min before baseline physiological parameters were recorded. After anesthetic administration, the onset time was recorded as the duration from injection to lateral recumbency. Physiological monitoring included electrocardiography (ECG), peripheral oxygen saturation (SpO_2_), and Temp, using a multiparameter monitor (CARESCAPE Monitor B650, GE HealthCare, Chicago, IL, USA). End-tidal carbon dioxide concentration (EtCO_2_) was monitored via a CO_2_ sidestream sampling line attached to nasal opening to ensure patient safety. This monitoring approach was based on preliminary trials, in which EtCO_2_ values remained within expected physiological ranges (35–45 mmHg). The study was conducted in a controlled room temperature maintained at 24–25 °C. Oxygen supply equipment and appropriately sized endotracheal tubes were prepared for immediate use to secure the airway and provide supplemental oxygen if necessary. Suction equipment and gauze were also prepared to manage potential airway obstruction caused by excessive salivation. Emergency drugs, including atropine, glycopyrrolate, epinephrine, and ephedrine, were readily available, and more than two experienced veterinarians were continuously present to monitor the animals and provide prompt intervention if required.

Physiological parameters, including HR, systolic arterial pressure (SAP), mean arterial pressure (MAP), diastolic arterial pressure (DAP), RR, SpO_2_, Temp, and withdrawal reflex were recorded at 5-min intervals. SpO_2_ measurement was initiated after 5 min. Temp measurement was also initiated after 5 min to minimize cardiovascular effects caused by excitement during measurement. The withdrawal reflex was assessed by applying mosquito forceps at the interdigital area until the first ratchet closed. A response was defined as any voluntary limb movement or withdrawal observed immediately after the application of the stimulus. The number of dogs exhibiting a response was recorded at each time point in all groups. The anesthetic time was defined as the time from lateral recumbency to the resumption of a sternal recumbency position. The analgesic time was defined as duration of absence of withdrawal reflex. The recovery quality was assessed based on a standardized scoring system (Table 1).

Humane endpoints were predefined: dogs were observed continuously for signs of severe distress, including cyanosis, abnormal respiratory effort, or apnea lasting more than 15 s. Monitoring was conducted every 5 min with continuous visual observation by veterinarians. If such signs were detected, immediate intervention (oxygen supplementation, airway management, or drug administration) was to be provided.

### 2.4. Statistical Analyses

The generalized estimated equation (GEE) method was employed to analyze group-wise comparisons, time-dependent changes, and interactions between groups and time points. Onset time, anesthetic time, analgesic time, and recovery scores were analyzed using the Kruskal–Wallis test. If a significant difference was identified, post hoc comparisons were performed using Mann–Whitney test. A *p*-value < 0.05 was considered statistically significant. The statistical analyses were performed using commercial software (SPSS 29, IBM Corp., Armonk, NY, USA).

## 3. Results

The six dogs included in this study had a body weight of 10.8 (8.9–13.1) kg. No abnormal values were identified in the physical examinations of all dogs before the experiment. HR, RR, SAP, MAP, DAP, SpO_2_, and Temp were analyzed statistically and plotted as graphs based on measurements taken up to 60 min.

### 3.1. Anesthesia Variables

All groups exhibited rapid induction, reaching lateral recumbency within 10 min following IM injection (Table 2). Although a trend toward a shorter onset time was observed with increasing medetomidine dose, the differences among the three treatment groups were not statistically significant. Similarly, anesthetic time tended to increase with higher medetomidine doses, yet no significant differences were detected. The Kruskal–Wallis test revealed a significant difference in analgesic time among the groups (*p* < 0.001). Post hoc analysis using the Mann–Whitney test further confirmed a significant difference between TZT and TZTM10 (*p* = 0.009), TZT and TZTM20 (*p* = 0.002), and TZTM10 and TZTM20 (*p* = 0.002). The median analgesic time was 0, 27.5, and 47.5 min in TZT, TZTM10, and TZTM20, respectively, demonstrating a dose-dependent prolongation with increasing medetomidine dose. Recovery quality scores were significantly higher in groups receiving medetomidine, as determined by the Kruskal–Wallis test (*p* = 0.002). Mann–Whitney post hoc analysis identified significant differences between TZT and TZTM10, as well as between TZT and TZTM20 (*p* = 0.002 for both). However, no significant difference was observed between TZTM10 and TZTM20 (Table 2).

### 3.2. Cardiorespiratory Variables

**Figure 1:**
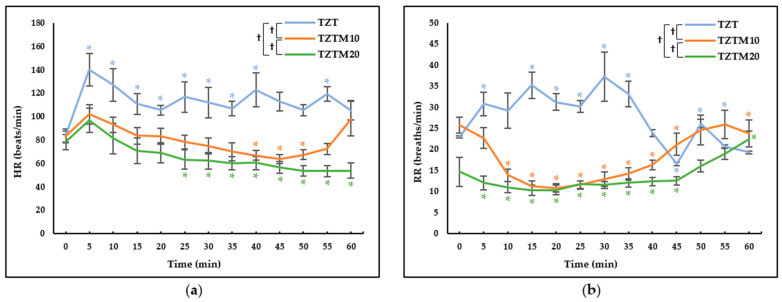
Heart rate (HR) and respiratory rate (RR) changes after intramuscular administration of 2.5 mg/kg of tiletamine, 2.5 mg/kg of zolazepam, and 4 mg/kg of tramadol prepared as a fixed-concentration solution and administered 0.1 mL/kg (TZT), with the addition of 10 µg/kg (TZTM10) or 20 µg/kg (TZTM20) of medetomidine in dogs. Data are presented as estimated mean ± standard error. * Significant differences from the baseline values within each group. † Significant differences between two groups. (**a**) Heart rate changes. The values show an increasing trend in the first 5 min, followed by a decrease; (**b**) Respiratory rate changes. In the groups that received medetomidine, the values remained generally low and gradually increased over time.

**Figure 2:**
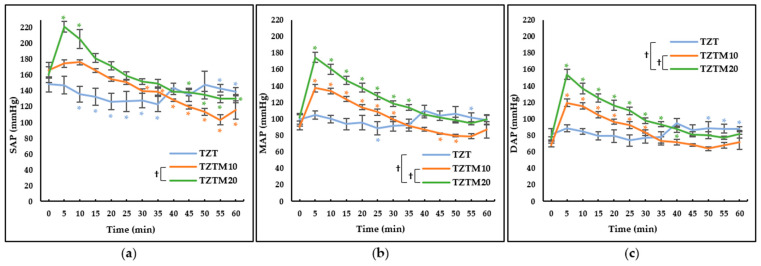
Blood pressure changes after intramuscular administration of 2.5 mg/kg of tiletamine, 2.5 mg/kg of zolazepam, and 4 mg/kg of tramadol prepared as a fixed-concentration solution and administered 0.1 mL/kg (TZT), with the addition of 10 µg/kg (TZTM10) or 20 µg/kg (TZTM20) of medetomidine in dogs. Data are presented as estimated mean ± standard error. * Significant differences from the baseline values within each group. † Significant differences between two groups. (**a**) Systolic arterial pressure (SAP) changes. It significantly differed between TZTM10 and TZTM20 (*p* < 0.001); (**b**) Mean arterial pressure (MAP) changes. It significantly differed between TZT and TZTM20 (*p* = 0.029) and between TZTM10 and TZTM20 (*p* < 0.001); (**c**) Diastolic arterial pressure (DAP) changes. It significantly differed between TZT and TZTM20 (*p* = 0.044) and between TZTM10 and TZTM20 (*p* < 0.001).

Significant differences in HR and RR were observed among groups, across time points, and in their interactions. Significant differences were particularly observed in HR values between TZT and TZTM10 (*p* < 0.001), TZT and TZTM20 (*p* < 0.001), and TZTM10 and TZTM20 (*p* = 0.015). Also, in RR values, significant differences were identified between TZT and TZTM10 (*p* < 0.001), TZT and TZTM20 (*p* < 0.001), and TZTM10 and TZTM20 (*p* = 0.002). In the group that did not receive medetomidine (TZT), HR initially increased and remained elevated over time. The groups that received medetomidine (TZTM10 and TZTM20) showed an increase at 5 min, but the initial rise was not pronounced, and the values remained close to the baseline until approximately 30 min, followed by a gradual decreasing trend (Figure 1a). In TZT, RR initially increased and then showed a decreasing trend starting at 30 min, whereas in TZTM10, it initially decreased and gradually increased over time (Figure 1b). In TZTM20, RR was generally maintained at a stable level (Figure 2b).

### 3.3. Blood Pressures

Significant differences were observed in SAP, MAP, and DAP among groups, across time points, and in their interactions. Significant differences were particularly noted in SAP between TZTM10 and TZTM20 (*p* < 0.001); in MAP between TZT and TZTM20 (*p* = 0.029) and between TZTM10 and TZTM20 (*p* < 0.001); in DAP between TZT and TZTM20 (*p* < 0.044) and between TZTM10 and TZTM20 (*p* < 0.001). In the TZT, which did not receive medetomidine, BP remained stable (Figure 2). Initially, BP rapidly increased in the medetomidine-treated groups (TZTM10 and TZTM20), followed by a gradual decrease as time progressed (Figure 2). An increase in BP was observed from around 40 min in the TZT, which may have been influenced by the poor recovery quality observed in this group (Table 1, Figure 2).

### 3.4. SpO_2_ and Temperature

**Figure 3:**
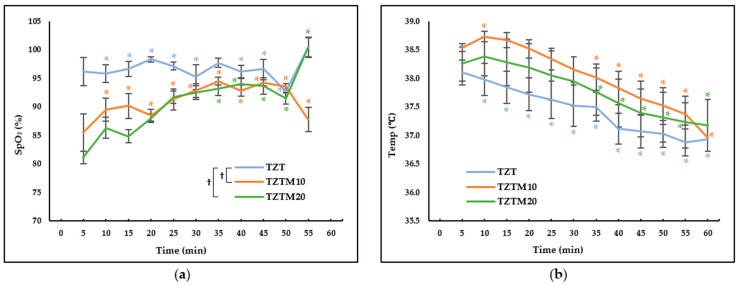
Peripheral oxygen saturation (SpO_2_) and body temperature (Temp) changes after intramuscular administration of 2.5 mg/kg of tiletamine, 2.5 mg/kg of zolazepam, and 4 mg/kg of tramadol prepared as a fixed-concentration solution and administered 0.1 mL/kg (TZT), with the addition of 10 µg/kg (TZTM10) or 20 µg/kg (TZTM20) of medetomidine in dogs. Data are presented as estimated mean ± standard error. * Significant differences from the baseline values within each group. † Significant differences between two groups. (**a**) SpO_2_ changes. It significantly differed between TZT and TZTM10 (*p* < 0.001) and between TZT and TZTM20 (*p* < 0.001); (**b**) Temp changes. It exhibited a gradual decrease across all groups.

Significant differences were observed in SpO_2_ and Temp across time points, and in group and time interactions. However, a significant difference was found only for SpO_2_ among groups, with no significant difference for Temp (*p* = 0.285). Significant differences in SpO_2_ were observed between TZT and TZTM10 (*p* < 0.001), and between TZT and TZTM20 (*p* < 0.001). SpO_2_ levels were lower in the medetomidine-treated groups (TZTM10 and TZTM20) than in the TZT but showed a gradual increasing over time (Figure 3). Clinical signs of hypoxemia, such as cyanosis or abnormal respiratory effort, were not observed in any dog during the procedures. No significant differences in Temp were found, though a general decreasing trend was observed in all groups. Although EtCO_2_ was monitored throughout the experiment, no abnormal values were noted in any group, and the measurements were not formally recorded.

Throughout the experiment, no dog required rescue intervention, such as oxygen supplementation, intubation, or suction for salivation. All animals maintained stable clinical conditions without adverse events that necessitated emergency management.

## 4. Discussion

This study hypothesized that the addition of medetomidine to a tiletamine-zolazepam-tramadol combination would result in dose-dependent changes in analgesic duration, recovery quality, and physiological stability. The results supported this hypothesis, demonstrating that increasing the medetomidine dose up to 20 µg/kg led to a marked prolongation of analgesic duration and improved in recovery scores. In addition, HR and RR were more stable in the medetomidine-treated groups compared to the control, suggesting enhanced autonomic regulation. These findings indicate that medetomidine contributes to a more effective and physiologically stable anesthetic protocol when administered intramuscularly in a dose-dependent manner.

The results of this study can be explained by the complementary pharmacological profiles of the agents used. Tiletamine, an N-methyl-D-aspartate (NMDA) receptor antagonist, provides dissociative anesthesia and analgesia but is associated with excitatory side effects such as muscle rigidity, seizure activity, and dysphoric recovery, particularly at higher doses [20]. Zolazepam, a benzodiazepine, counteracts these effects through muscle relaxation and anticonvulsant activity, while also contributing to amnesia [21,22]. This combination has an onset of action ranging from 5 to 12 min, with a duration of approximately 30 to 60 min following IM administration in dogs [23]. Tramadol, a centrally acting opioid structurally related to codeine and morphine, has been investigated as a potential analgesic in veterinary medicine [2,10]. Its action is primarily mediated through μ-opioid receptor activation by both the parent compound and its metabolites. Although tramadol is rapidly and almost completely absorbed after IM administration, with systemic availability comparable to IV injection. The differences in onset and duration of action between IM and IV routes were minimal and likely clinically insignificant, supporting the viability of IM administration in dogs [24]. However, its affinity for μ- and δ-opioid receptors is low, and even weaker affinity for the κ-opioid receptor subtype, which results in limited analgesic effect [19,25]. Consequently, its analgesic efficacy in dogs remains controversial and is generally considered limited. In this study, tramadol was not expected to serve as a primary analgesic but rather included as an adjunct to support multimodal analgesia in combination with agents possessing different mechanisms of action. Medetomidine induces sedation and visceral analgesia by activating α_2_-adrenergic receptors, leading to central nervous system (CNS) depression. Additionally, it promotes muscle relaxation and has a relatively short duration of action, making it suitable for procedures lasting less than 1 h. Although its analgesic effects are not strictly dose-dependent, this study found that increasing the dose was associated with prolonged analgesic duration and improved recovery quality. Its effect can be reversed with an α_2_-adrenergic receptor antagonist, such as atipamezole [26]. However, due to its effects on the cardiovascular system, particularly peripheral vasoconstriction, caution is required in animals with pre-existing cardiovascular conditions, and continuous monitoring is necessary.

Combining dissociative agents, benzodiazepines, opioids, and α_2_-adrenergic agonists allows for dose reduction of individual drugs while effectively producing analgesia, unconsciousness, and muscle relaxation [10,11]. In this experiment, a rapid onset time of approximately 5 min was observed in all groups receiving tiletamine-zolazepam and tramadol, regardless of the administration of medetomidine (Table 2). Although the difference was not statistically significant, the onset time tended to decrease as the medetomidine dose increased. Although no statistically significant difference was observed in anesthetic time, the trend of prolonged anesthetic time was also noted with increasing medetomidine dosage. Among the measured variables, analgesic time showed the most significant difference with varying medetomidine doses (Table 2). Despite the presence of dissociative agents and opioids, TZT exhibited a limited analgesic effect in response to noxious stimuli. However, as the medetomidine dose was increased to 10 and 20 µg/kg, a significant prolongation of analgesic duration was observed in TZTM10 and TZTM20. Notably, TZTM20 exhibited an analgesic time of nearly 50 min (Table 2). A significant difference in recovery quality was observed between the group without medetomidine (TZT) and the groups with medetomidine (TZTM10 and TZTM20). The recovery quality score was higher in the groups that received medetomidine. These findings suggest that the addition of medetomidine mitigates adverse effects commonly seen with dissociative anesthetics, such as rough recoveries characterized by excitement, dysphoria, or ataxia. The results further demonstrate that a medetomidine dose of 20 µg/kg provides the most pronounced enhancement in analgesic effects (Table 2).

The effects of the medetomidine-containing combination were reflected in the cardiorespiratory variables. In all groups, HR increased at approximately 5 min post-injection, likely due to sympathetic stimulation from the dissociative agent. In TZT, this increase was pronounced and remained elevated above the baseline value, indicating sustained sinus tachycardia. Since the duration of tiletamine-zolazepam is between 30 and up to 60 min [23], HR showed a gradual decline over time but remained above the baseline value. In contrast, the medetomidine-treated groups (TZTM10 and TZTM20) showed a brief initial rise in HR at 5 min, followed by a gradual return to baseline by 10 min (Figure 1a). Subsequently, HR continued to decrease, with bradycardia becoming evident at 15 min in TZTM10 and at 25 min in TZTM20. This delayed onset may be explained by the interplay between the early sympathomimetic effects of tiletamine and the vasoconstrictive action of medetomidine. It is likely that the initial sympathetic dominance temporarily suppressed the baroreceptor reflex. As the dissociative effect diminished, medetomidine-induced hypertension elicited a baroreceptor-mediated response, resulting in reflex bradycardia [26]. In TZT, RR increased similarly to HR, which is presumed to result from sympathetic stimulation, displaying an unstable pattern. However, in TZTM10 and TZTM20, RR initially decreased and remained relatively stable with regular RR interval throughout the observation period (Figure 1b). α_2_-adrenergic agonists are known to have minimal effects on the respiratory system in healthy animals [26]. However, the results of this study suggest that they may also help stabilize RR fluctuations induced by other anesthetic agents.

The administration of α_2_-adrenergic agonists induces a biphasic, dose-dependent BP response, characterized by an initial increase followed by normalization or slightly decline [26]. Initially, activation of α_2_-adrenergic agonists in the peripheral vasculature induces peripheral vasoconstriction, leading to an increase in BP as an immediate cardiovascular response. Consequently, the baroreceptor reflex is triggered, leading to sinus bradycardia [26]. Subsequently, as the peripheral vasoconstrictive effect diminishes, BP gradually decreases to baseline or slightly lower values. However, the drug’s action on presynaptic α_2_-adrenergic agonists in the CNS induces a prolonged reduction in sympathetic nervous system tone, resulting in the sustained presence of bradycardia [26]. This biphasic cardiovascular response underscores the need for a thorough understanding of its mechanisms to ensure safe and effective use of these agents. In this study, the initial rapid increase in BP was evident in TZTM10 and TZTM20, where medetomidine was administered (Figure 2). Based on MAP, BP gradually decreased to baseline levels after approximately 35 min, and in TZTM20, it remained stable around the baseline value (Figure 2b). In the TZT, BP remained relatively stable, with an increase observed around the 40-min mark (Figure 2), likely from arousal from anesthesia (Table 2).

Studies have reported that IV administration of tiletamine-zolazepam caused more pronounced transient hypoxemia than IM administration [27]. In this study, IM administration was associated with relatively stable SpO_2_ levels in the TZT group (Figure 3a). In contrast, in the medetomidine-treated groups, SpO_2_ was initially measured at a lower level. Medetomidine induces peripheral vasoconstriction and enhances oxygen extraction in peripheral tissues, resulting in increased venous desaturation [28]. In this study, the observation that SpO_2_ levels decreased in a medetomidine dose-dependent manner (Figure 3a) suggests that the previously explained mechanism contributed to this reduction in SpO_2_. The use of α_2_-adrenergic agonists induces peripheral vasoconstriction, potentially weakening peripheral pulses despite central hypertension and reducing cutaneous blood flow, which may compromise pulse oximetry [26,28,29]. Clinicians should be aware that a decrease in SpO_2_ following α_2_-adrenergic agonist administration does not necessarily reflect a reduction in PaO_2_ or the presence of hypoxemia. Nonetheless, in clinical settings where endotracheal intubation is not performed, supplemental oxygen should be considered to reduce the risk of hypoxemia, especially when α_2_-adrenergic agonists are used and pulse oximetry reliability may be compromised. Among the measured parameters, Temp was the only one without significant differences between groups. Although a transient increase was observed in the medetomidine-treated groups initially, a more pronounced decreasing trend over time was evident across all groups. While α_2_-adrenergic agonists exert a direct depressant effect on the thermoregulatory center, peripheral vasoconstriction minimizes heat loss, potentially contributing to Temp maintenance [26]. As a result of these characteristics, a significant decrease in Temp over time was observed from the early stages in the TZT group, whereas in TZTM10 and TZTM20, no significant reduction in Temp was detected until approximately 30 min (Figure 3b).

As high-dosage tiletamine-zolazepam products are unavailable in South Korea, an alternative formulation containing 125 mg of each component was used. This tiletamine-zolazepam formulation was combined with 50 mg/mL tramadol, with or without 1 mg/mL medetomidine, to prepare the study anesthetic. The anesthetic mixture was administered at 0.1 mL/kg, containing 2.5 mg/kg of tiletamine and zolazepam each and 4 mg/kg of tramadol in the TZT. The medetomidine dose was 10 µg/kg in TZTM10 and increased to 20 µg/kg in TZTM20.

Butorphanol is a mixed agonist-antagonist opioid that exhibits high-affinity antagonism at μ-opioid receptors while selectively activating κ-opioid receptors [19,30,31,32]. As a result of this pharmacological profile, it demonstrates a ceiling effect, thereby limiting its analgesic efficacy. Furthermore, since it antagonizes the μ-opioid receptor, which plays a key role in analgesia, it inevitably reduces the effects of endogenous opioids, such as endorphins, dynorphins, and endomorphins [33]. In contrast, tramadol has a relatively low affinity for opioid receptors; however, it is known to be effective not only for acute pain but also for chronic pain [19,25,34]. Additionally, analgesic efficacy of tramadol is enhanced when used in combination with other analgesic agents [34]. This study evaluated an anesthetic combination in which tramadol replaced butorphanol, with medetomidine administered at varying doses. The results demonstrated that medetomidine helped stabilize RR (Figure 1b), facilitated a smooth recovery from anesthesia (Table 2), and may have contributed to Temp regulation (Figure 3b). Notably, increasing the dose of medetomidine was associated with a faster onset, a prolonged anesthesia duration of approximately 60 min, and a significant extension of analgesic duration (Table 2). This prolongation of analgesia can be attributed to the combined effects of different drug classes, including NMDA antagonists, α_2_-adrenergic agonists, and opioid receptor agonists, which modulate pain processing through distinct mechanisms. Consequently, their combination enhances analgesic efficacy, a concept known as multimodal analgesia [19]. In the TZTM20, where medetomidine was administered at 20 μg/kg, profound unconsciousness, muscle relaxation, and analgesic effects were observed. Additionally, stable respiration and a smooth recovery process were noted. This combination produced anesthetic and analgesic effects lasting approximately 60 min in this laboratory setting. However, clinical studies are needed to confirm its efficacy in specific surgical procedures, as analgesic requirements vary with the type and severity of surgical stimulation. In any case, its cardiovascular effects warrant careful monitoring when applied clinically. A significant initial increase in BP was observed (Figure 2), which may pose risks not only to patients with underlying cardiovascular diseases but also by elevating intraocular and intracranial pressure. Therefore, its use should be carefully considered in patients with related conditions. Additionally, increased BP may lead to greater blood loss; therefore, this combination should be used with caution during neutering procedures, particularly in engorged blood vessels. Potential complications should be anticipated, and in the event of a fatal complication, immediate intervention is required. Therefore, facilities should be adequately equipped for anesthetic administration, patient monitoring, and emergency response [35]. If necessary, the effects of α_2_-adrenergic agonists can be reversed using antagonists such as atipamezole.

Despite its valuable findings, this study has several limitations that should be acknowledged. First, the study involved six Beagle dogs that were repeatedly assigned to treatment groups, resulting in a limited sample size of six per group. Although this design permitted statistical analysis, missing data at certain time points necessitated the use of nonparametric methods. A priori sample size calculation was not conducted; instead, the number of subjects was determined by referencing previous studies with similar objectives and designs. While this approach provided power for within-subject comparisons, the results may have limited generalizability, as they may not directly apply to other breeds, ages, or physiological states. Second, a standard control group was not included, which restricts comparisons with established clinical protocols. Third, PaO_2_ was not directly measured. Although SpO_2_ was monitored, its accuracy may have been affected by peripheral vasoconstriction associated with α_2_-adrenergic agonists, and may not reflect true oxygenation. Future studies utilizing arterial blood gas analysis would provide a more definitive evaluation of oxygenation status. Similarly, although the respiratory rate remained stable, minute volume was not measured, highlighting the need to assess PaCO_2_ for a more comprehensive evaluation of ventilation. In addition, this study was conducted under controlled laboratory conditions without actual surgical procedures, which may limit the clinical relevance of the results. Analgesia was assessed solely through the withdrawal reflex to avoid repeated stimulation; however, acute autonomic responses—such as changes in HR, BP, or RR—were not analyzed in real time. As the study focused on general anesthetic effects, synchronized physiological monitoring around nociceptive testing was not included. Future research should incorporate real-time assessments to better characterize analgesic effect. Although HR, BP, and RR were continuously monitored, standard clinical indicators of anesthetic depth—such as palpebral reflex, jaw tone, and eye position—were omitted. This decision was made to minimize interference with physiological baselines, but it remains a limitation. Also, endotracheal intubation was intentionally omitted to reflect the conditions in which IM anesthesia is typically used—such as in shelter programs or mobile clinics where inhalant delivery systems and airway devices may not be available. While this decision was made to reflect clinical reality, it inevitably limited the ability to monitor ventilation and confirm anesthetic depth. Whenever feasible, airway protection and supplemental oxygen should be implemented in clinical practice. Finally, individual patient variability, surgical stress, and environmental factors in clinical settings may influence anesthetic outcomes. Further validation across a broader range of breeds, ages, and procedures is warranted to improve the generalizability of these findings.

## 5. Conclusions

This study evaluated the anesthetic and cardiorespiratory effects of three IM anesthetic protocols combining tiletamine-zolazepam, tramadol, and varying doses of medetomidine in dogs. The findings indicate that increasing the medetomidine dose up to 20 µg/kg resulted in a dose-dependent prolongation of analgesic duration, improved recovery quality, and stable respiratory function. Notably, the combination containing 20 µg/kg medetomidine (TZTM20) provided the most pronounced benefits, including prolonged analgesia, smoother recovery, and adequate anesthetic duration of approximately 60 min. The incorporation of multimodal analgesia by combining dissociative anesthetics, benzodiazepines, opioids, and α_2_-adrenergic agonists contributed to enhanced anesthetic efficacy and stability. While the addition of medetomidine improved analgesia and recovery, it also induced transient hypertension due to its peripheral vasoconstrictive effects. Although this biphasic cardiovascular response stabilized over time, careful monitoring is required, particularly in patients with pre-existing cardiovascular conditions or those at risk of increased intraocular or intracranial pressure. These findings support the use of this anesthetic combination for procedures requiring approximately one hour of anesthesia while maintaining respiratory stability. However, the study was conducted under controlled laboratory conditions without surgical intervention, and variations in patient physiology, surgical stress, and environmental factors may influence outcomes in clinical practice. Future research should validate these findings in diverse clinical scenarios, including different breeds, age groups, and surgical settings, to refine and optimize anesthetic protocols for veterinary applications.

## Figures and Tables

**Table 1:** The scoring system used to evaluate the recovery quality of the dogs.

Recovery Score	Description
Poor (Score 1)	Persistent disorientation, hyperrigidity, hypersalivation, howling, multiple attempts to stand, uncoordinated gait, dragging, and wandering.
Fair (Score 2)	Mild disorientation, multiple attempts to stand, transient ataxia, relaxed posture, absence of excessive salivation.
Good (Score 3)	Minimal disorientation, few attempts to stand, well-coordinated gait, relaxed demeanor.
Excellent (Score 4)	No disorientation, successful first attempt to stand, calm and alert, responsive to commands.

**Table 2:** Onset (time of injection to lateral recumbency), anesthetic (duration of lateral recumbency), analgesic time (duration of absence of withdrawal reflex), and recovery quality after intramuscular administration of 2.5 mg/kg of tiletamine, 2.5 mg/kg of zolazepam, and 4 mg/kg of tramadol prepared as a fixed-concentration solution and administered 0.1 mL/kg (TZT), with the addition of 10 µg/kg (TZTM10) or 20 µg/kg (TZTM20) of medetomidine in dogs.

Variables	TZT (*n* = 6)	TZTM10 (*n* = 6)	TZTM20 (*n* = 6)
Onset time (min)	6.0 (3–8)	5.5 (3–9)	4.5 (3–7)
Anesthetic time (min)	41.5 (25–85)	58.5 (50–77)	65.0 (55–85)
Analgesic time (min) *	0 (0–15)	27.5 (10–35)	47.5 (40–55)
Recovery quality (score) **	2 (1–2)	3 (3–4)	4 (3–4)

Data are presented as median (range). * Significant difference was identified using the Kruskal–Wallis test (*p* < 0.001). Post hoc analysis using the Mann–Whitney test identified significant differences, with *p*-values of 0.009, 0.002, and 0.002 for comparisons between TZT and TZTM10, TZT and TZTM20, and TZTM10 and TZTM20, respectively. ** Significant differences in recovery quality scores were detected using the Kruskal–Wallis test (*p* = 0.002). ** Post hoc analysis using the Mann–Whitney test revealed significant difference (*p* = 0.02) for comparisons between TZT and TZTM10 and between TZT and TZTM20.

## Data Availability

The raw data supporting the conclusion of this article will be made available by the authors on request.
